# miR-375 gene dosage in pancreatic β-cells: implications for regulation of β-cell mass and biomarker development

**DOI:** 10.1007/s00109-015-1296-9

**Published:** 2015-05-28

**Authors:** Mathieu Latreille, Karolin Herrmanns, Neil Renwick, Thomas Tuschl, Maciej T. Malecki, Mark I. McCarthy, Katharine R. Owen, Thomas Rülicke, Markus Stoffel

**Affiliations:** Institute of Molecular Health Sciences, Swiss Federal Institute of Technology (ETH Zurich), Otto-Stern Weg. 7, 8093 Zurich, Switzerland; MRC Clinical Sciences Centre, Imperial College London, Hammersmith Hospital Campus, Du Cane Road, London, W12 0NN UK; Howard Hughes Medical Institute, Laboratory of RNA Molecular Biology, The Rockefeller University, 1230 York Avenue, Box 186, New York, NY 10065 USA; Department of Metabolic Diseases, Jagiellonian University Medical College, Krakow, Poland; University Hospital, Krakow, Poland; Oxford Centre for Diabetes, Endocrinology and Metabolism, Churchill Hospital, University of Oxford, Old Road, Headington, Oxford, OX3 7LJ UK; Wellcome Trust Centre for Human Genetics, University of Oxford, Roosevelt Drive, Oxford, OX3 7BN UK; Oxford NIHR Biomedical Research Centre, Churchill Hospital, Old Road, Headington, Oxford, OX3 7LJ UK; Institute of Laboratory Animal Science, University of Veterinary Medicine, Vienna, Austria; Faculty of Medicine, University of Zurich, Zurich, Switzerland

**Keywords:** MiRNA-375, Pancreatic β-cells, Biomarker, Diabetes, β-cell mass

## Abstract

**Abstract:**

MicroRNAs play a crucial role in the regulation of cell growth and differentiation. Mice with genetic deletion of miR-375 exhibit impaired glycemic control due to decreased β-cell and increased α-cell mass and function. The relative importance of these processes for the overall phenotype of miR-375KO mice is unknown. Here, we show that mice overexpressing miR-375 exhibit normal β-cell mass and function. Selective re-expression of miR-375 in β-cells of miR-375KO mice normalizes both, α- and β-cell phenotypes as well as glucose metabolism. Using this model, we also analyzed the contribution of β-cells to the total plasma miR-375 levels. Only a small proportion (≈1 %) of circulating miR-375 originates from β-cells. Furthermore, acute and profound β-cell destruction is sufficient to detect elevations of miR-375 levels in the blood. These findings are supported by higher miR-375 levels in the circulation of type 1 diabetes (T1D) subjects but not mature onset diabetes of the young (MODY) and type 2 diabetes (T2D) patients. Together, our data support an essential role for miR-375 in the maintenance of β-cell mass and provide in vivo evidence for release of miRNAs from pancreatic β-cells. The small contribution of β-cells to total plasma miR-375 levels make this miRNA an unlikely biomarker for β-cell function but suggests a utility for the detection of acute β-cell death for autoimmune diabetes.

**Key messages:**

- Overexpression of miR-375 in β-cells does not influence β-cell mass and function.
- Increased α-cell mass in miR-375KO arises secondarily to loss of miR-375 in β-cells.
- Only a small proportion of circulating miR-375 levels originates from β-cells.
- Acute β-cell destruction results in measurable increases of miR-375 in the blood.
- Circulating miR-375 levels are not a biomarker for pancreatic β-cell function.

**Electronic supplementary material:**

The online version of this article (doi:10.1007/s00109-015-1296-9) contains supplementary material, which is available to authorized users.

## Introduction

Pancreatic α- and β-cells are the main cell types regulating glucose metabolism through the secretion of glucagon and insulin, respectively. In adult mice, β-cell mass is mostly determined through a delicate balance between β-cell replication and apoptosis. Maintenance of proper β-cell mass is required for maintaining glucose homeostasis, and an inability to renew pancreatic β-cell mass results in the development of diabetes. Autoimmune destruction of insulin-producing cells underlies the pathophysiology of type 1 diabetes (T1D) [1], whereas chronic metabolic stress linked to obesity and insulin resistance results in a gradual impairment of pancreatic function, demise of β-cells, and ultimately causes type 2 diabetes (T2D) [2, 3]. Many regenerative therapeutic strategies have been envisaged to increase β-cell mass or delay its decline in T1D and T2D patients; however, most of these interventions showed poor efficacy [4].

miRNAs are a class of small regulatory RNAs that repress the expression of selected target mRNAs and confer robustness and stability to biological networks in physiological and pathological conditions [5]. How post-transcriptional regulation by miRNAs impinges on the regulation of islet endocrine cell mass still remains to be completely understood. miR-375 represents the most highly transcribed miRNA gene in β-cells with significant expression in other endocrine organs such as the pituitary, adrenal glands, skin, and intestine [6–9]. Global miR-375 gene inactivation in mice leads to overt diabetes due in part to decreased β-cell mass [10]. Several negative regulators of cell growth are induced in miR-375KO islets and underlie the anti-proliferative effects in pancreatic β-cells. The importance of miR-375 in obesity-induced β-cell mass expansion was demonstrated in miR-375 and leptin double deficient (miR-375KO; *ob/ob*) mice, which exhibit impaired β-cell proliferation and profoundly impaired compensatory β-cell hypertrophy [10]. Noteworthy is the increased α-cell mass and circulating glucagon levels found in miR-375KO mice, which induce augmented hepatic glucose production and, together with the decreased β-cell function, further exacerbates glycemic control [10]. However, the relative importance of α- and β-cell defects in the diabetic phenotype of global miR-375KO mice still remains to be determined.

In recent years, miRNAs have been detected in extracellular fluids, including blood, saliva, breast milk, and other body secretions [11, 12]. miRNAs are secreted by cells via exosomes and travel in the circulation attached to high-density lipoprotein particles, where they can be taken up by recipient cells cultured in vitro through receptor-mediated endocytosis [13, 14]. Alternative routes of miRNA secretion through microvesicles have also been described [15, 16]. Several studies have suggested that they may serve as serum biomarkers because of their remarkable stability in blood and characteristic expression in different tissues and disease states [17]. miR-375 has been shown to be increased in the plasma of two murine models with profound pancreatic β-cell death, NOD, and streptozotocin (STZ)-treated mice [18]. However, whether circulating miR-375 levels derive from β-cells in these models and if miR-375 levels are modulated in diabetes remains to be determined.

To further dissect the role of miR-375 gene dosage in the regulation of pancreatic endocrine cell mass and glucose metabolism, we generated and characterized transgenic mice overexpressing miR-375 selectively in pancreatic β-cells (named “Tg375”). This model was used to rescue miR-375 expression in pancreatic β-cells of global miR-375KO animals and to investigate miR-375’s specific role in the maintenance of β-cell function and regulation of blood glucose homeostasis in vivo. Furthermore, we used these mice to determine the overall contribution of β-cells to steady state plasma miR-375 levels and contrast these to levels measured in different types of murine and human diabetes.

## Materials and methods

### Experimental animals

Animal models were housed in a pathogen-free animal facility at the Institute of Molecular Systems Biology, ETHZ. The animals were maintained on a 12-hr light/dark cycle and fed a standard rodent chow. All animal experiments were approved by the kantonale Veterinäramt Zürich. miR-375KO mice were maintained on a pure C57BL/6N background and described previously [10].

### Generation of miR-375 transgenic mice

Transgenic mice expressing miR-375 under the regulation of the rat insulin promoter (RIP) were generated by inserting a 141-bp fragment encompassing the genomic murine pre-miR-375 sequence into *KpnI* and *HindII* sites of pCRII-RIP generating pCRII-RIP-miR-375. A 1.1-kb DNA fragment generated upon digestion of pCRII0-RIP-miR-375 with *NsiI* and containing the pRIP-miR-375 transgene was injected into male pronuclei of C57BL/6N zygotes to generate “Tg375” transgenic mice. Two transgenic founder lines, designated as B6N-Tg(Rip-375)416; 417Biat, were characterized and displayed similar expression levels of miR-375 and metabolic phenotypes. All mice were maintained on a pure C57BL/6N background. Tg375 mice were genotyped using the following primers: 5′-GCAAGCAGGTATGTACTCTCCAG-3′ and 5′-AACGCTCAGGTCCGGTTT GTGCGAG-3′.

### Intraperitoneal glucose, insulin, and pyruvate tolerance tests

Blood glucose was measured using a Contour glucometer (Bayer). For intraperitoneal glucose tolerance tests (IPGTT), overnight fasted (13 h) mice were injected with D-glucose solution at 2 g/kg. For insulin tolerance tests (ITT), animals were injected with 0.75 U/kg body weight of a 5 × 10^−2^ U/ml insulin solution after a 6-h fasting period. For intraperitoneal pyruvate tolerance test (PTT), mice were injected with 2 g/kg in overnight fasted mice. Blood glucose was measured using a Contour glucometer (Bayer), insulin was measured by ELISA (Chrystal Chem), and glucagon levels were determined by EIA (Phoenix Pharmaceuticals). Streptozotocin was prepared in 100 mM sodium citrate pH 4.5 at a concentration of 7.5 mg/ml and administered once i.p. in 5-h fasted mice at a dose of 150 mg/kg.

### Islet secretion assays

Islet secretion studies were performed on size-matched islets following collagenase digestion and overnight culture in a RPMI 1640 medium, 5.5 mM glucose supplemented with 10 % heat-inactivated FBS, 2 mM L glutamine, 100 IU/ml penicillin, and 100 μg/ml streptomycin. Islet were incubated in Dulbecco’s PBS-Hepes-BSA buffer containing 1 mM glucose for 1 h before being transferred to Dulbecco’s buffer containing 3.3 and 16.7 mM glucose solutions for 30 min for static incubations.

### Morphometric analysis and miRNA FISH

Pancreata were fixed in 4 % paraformaldehyde and embedded in paraffin before sectioning to a thickness of 8 μm. For islet α- and β-cell mass analysis, five sections at least 180 μm apart were taken from each mouse (at least three mice per group), processed in immunofluorescence with anti-insulin (Sigma) and anti-glucagon antibodies (Millipore), and counterstained with DAPI. Pancreatic sections were scanned entirely using a 10× objective of a Zeiss AxioVert 200 microscope, and the images were recorded and assembled by AxionVision 4.6.3 software. The fraction of the insulin or glucagon positive areas were determined using NIH ImageJ software (http://rsbweb.nih.gov/ij/download), and finally, the mass was calculated by multiplying this fraction by the initial pancreatic wet weight. miRNA fluorescence in situ hybridization (FISH) was performed as described previously [19]. The miR-375 probe was synthesized with a linker that enabled conjugation of six biotin moieties: 5′-AGCCGaaCGaAcaaA-(L)3-B-L-B-L-B-L-B-N-B-(B-CPG), where uppercase letters indicate DNA nucleotides, lowercase letters indicate LNA modification, L represents spacer 18 (GlenResearch, catalog no. 10-1918-02), B represents protected biotinLC serinol (GlenResearch, catalog no. 10-1995-02), and B-CPG represents 3′-protected biotinLC serinol CPG (GlenResearch, catalog no. 20-2995-10).

### RNA isolation and miRNA quantification in plasma

RNA was isolated from pancreatic islets using Trizol reagent (Invitrogen) according to the manufacturer’s protocol. RNA was subjected to DNaseI treatment with the DNA-free kit (Invitrogen). RNA was reverse transcribed using a High Capacity cDNA Reverse Transcription kit (Applied Biosystems). Quantitative PCR was performed by Roche 384 real-time PCR machine and using Light Cycler^®^ 480 SYBR Green Master (Roche). miRNA levels were measured using the TaqMan microRNA Assays (Applied Biosystems), and the results were normalized to U6 RNA. Circulating and islet supernatant miRNA levels were determined using a spike-in protocol and the data analyzed as described in [20]. Briefly, 240 ng of the carrier MS2 RNA and 25 pmol of *Caenorhabditis elegans* miR-39 were spiked in Trizol before addition to each sample (50 μl plasma, 200 μl supernatant of cultured islets) and subjected to chloroform extraction and RNA isolation using the miRNeasy isolation kit (Qiagen). RNA was recovered in 30 μl of distillated water, and 3 μl was used in the reverse transcription reactions. Absolute miRNA quantification was performed by reverse transcription of serial dilutions of synthetic oligonucleotides with sequence to mature mmu-miR-375 (5′-UUUGUUCGUUCGGCUCGCGUGA-3′), mmu-miR-16 (5′-UAGCAGCACGUAAAUAUUGGCG-3′), and mmu-miR-194 (5′-UGUAACAGCAACUCCAUGUGGA-3′).

### Patient information

Subjects were recruited from the UK and Poland with informed consent prior to their inclusion in the study and approved by the ethics committee. UK subjects comprised subjects with HNF1α-MODY3 (*n* = 45), T1D (*n* = 38), T2D (*n* = 58), and no diagnosed metabolic disease or healthy controls (*n* = 51). Subjects with T1D or T2D were selected from the Young Diabetes in Oxford (YDX) study comprising individuals diagnosed with diabetes at ≤45 years of age. T2D was defined as follows: C-peptide positive, no requirement for permanent insulin within 3 months of diagnosis, absence of glutamic acid decarboxylase (GAD) antibodies. T1D was defined as a permanent insulin treatment since diagnosis, with evidence of severe β-cell dysfunction (C-peptide ≤ 0.2 nmol/l) and/or positive GAD antibodies (>14 WHO units/ml). Controls were normoglycaemic individuals aged 30–50 years from the Oxford Biobank. Clinical features of HNF1α and T2D subjects are found in Supplementary Table 1. Both groups of diabetics were on glucose-lowering treatment.

### Statistical analysis

Numerical values are reported as mean ± s.e.m. Unpaired Student’s *t* test was used for comparisons with two groups and ANOVA with Bonferroni post-test for comparisons of three or more groups. A *p* value <0.05 was considered as significant.

## Results

### Transgenic mice overexpressing miR-375 in β-cells have normal glucose tolerance

**Fig. 1:**
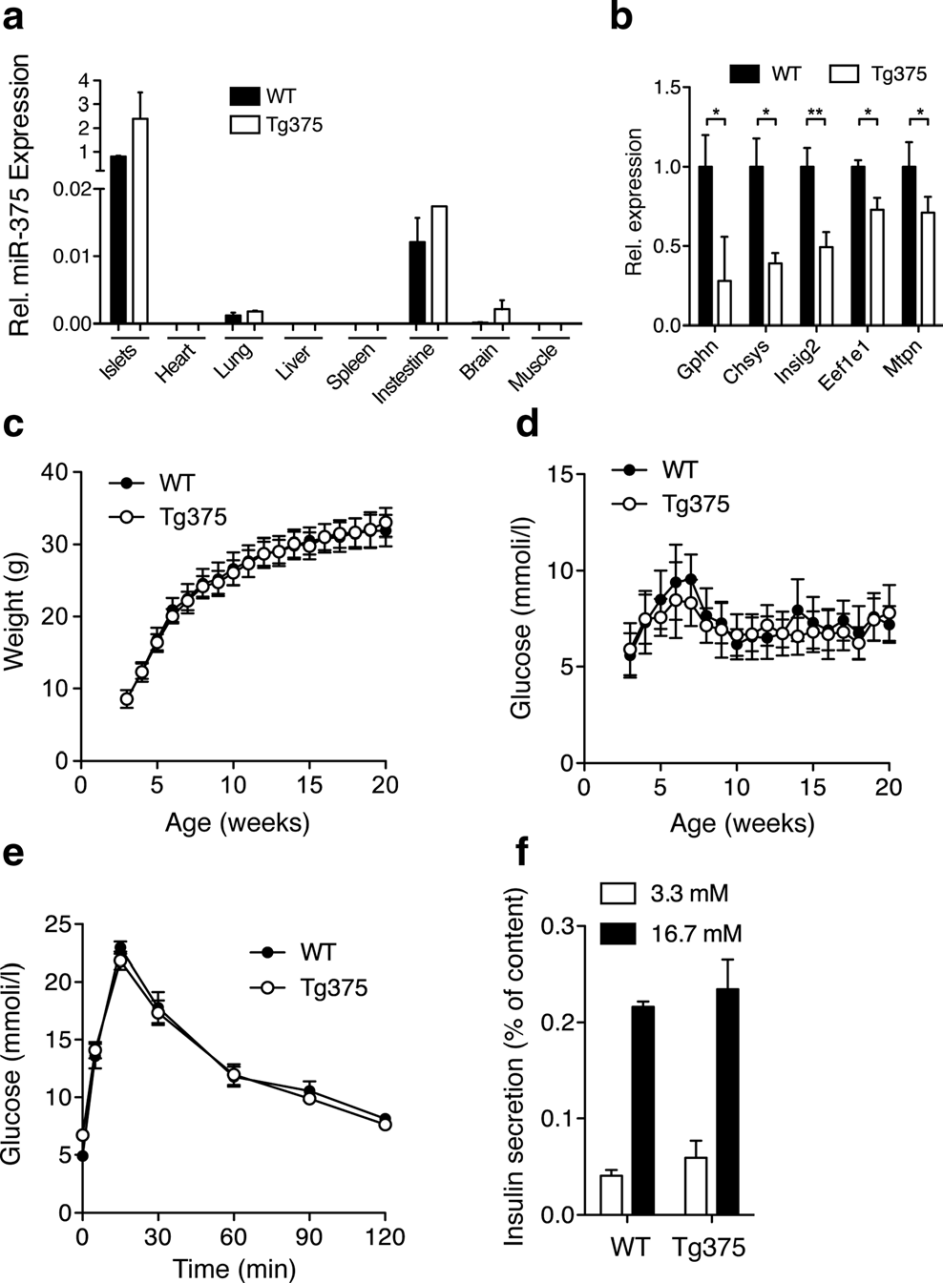
Metabolic characterization of β-cell-specific miR-375 transgenic mice. **a** Relative miR-375 expression in islets and indicated organs of male wildtype (WT) and Tg375 mice at 12 weeks of age (*n* = 5 for islets, *n* = 2 for all other tissues). **b** Relative expression of validated miR-375 targets in islets of male Tg375 mice and WT controls at 12 week of age. **c** Body weight of Tg375 (*white circles*) and WT (*black circles*) mice (*n* = 10). **d** Ad libitum-fed blood glucose levels in Tg375 and control littermate mice (*n* = 10). **e** Intraperitoneal Glucose Tolerance Test (IPGTT; 2 g/kg) in overnight fasted Tg375 (*white circles*) and WT (*black circles*) mice at 10 weeks of age (*n* = 11). **f** Static insulin secretion performed with 10-week-old control and Tg375 islets (*n* = 5) at 3.3 mmol/l (*white bars*) and 16.7 mmol/l (*black bars*) glucose concentrations. All data shown are mean ± s.e.m., except for panel **c** and **d** where s.d. is shown. *p < 0.05, **p < 0.01

To further characterize the β-cell growth promoting activity of miR-375, we generated transgenic miR-375 mice in which the pre-miR-375 was cloned downstream of the insulin promoter (referred to as Tg375). Two founder lines were derived from pronuclei microinjections, and both lines displayed ≈2-fold overexpression of miR-375 in pancreatic islets, without “leakage” in other organs such lung, spleen, muscle, colon, kidney, or heart, except minor escape in the brain, consistent with leakage of the insulin promoter in selected hypothalamic neurons (Fig. 1a). We confirmed that expression of validated mRNA targets of miR-375 such as Gphn, Chsys, Insig2, Mtpn, and Eef1e1 were downregulated in Tg375 islets (Fig. 1b) [10]. Surprisingly, metabolic characterization of Tg375 did not reveal significant changes in weight, blood glucose, glucose tolerance, or pancreatic endocrine function as compared to control littermate mice (Figs. 1c–f, 3, and 4). These results indicate that increased miR-375 gene dosage in pancreatic β-cells of mice does not alter pancreatic endocrine cell composition and glucose tolerance.

### Selective re-expression of miR-375 in β-cells of miR-375KO mice restores normal glycemic control

**Fig. 2:**
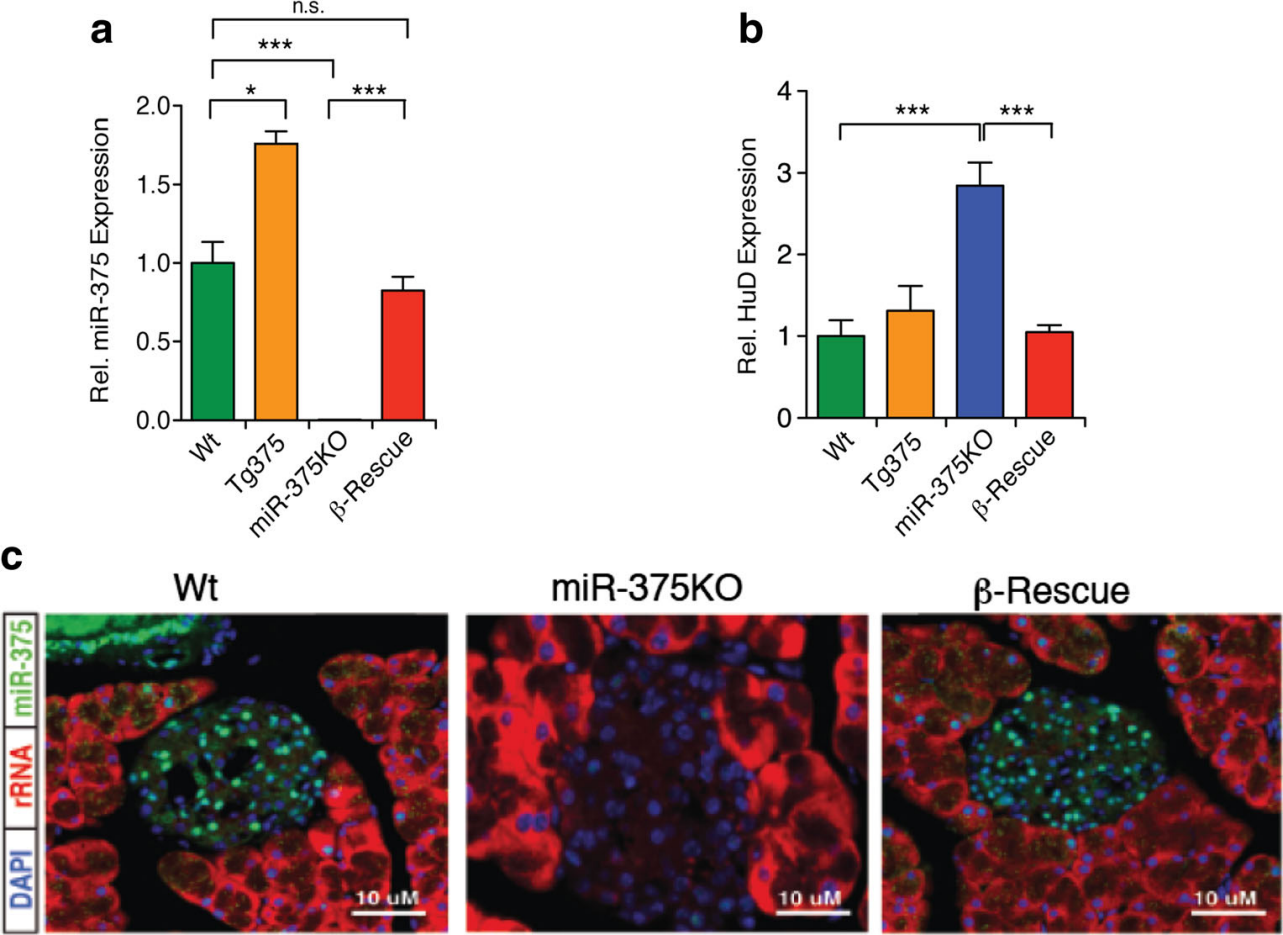
Functional characterization of miR-375KO mice with selective re-expression of miR-375 in pancreatic β-cells. **a** Relative miR-375 expression in islets of male WT, Tg375, miR-375KO, and β-Rescue mice at 12 weeks of age (*n* = 4–5). Data expressed as fold-change over WT controls. **b** Relative expression of the miR-375 target HuD in islets of male WT, Tg375, miR-375KO, and β-Rescue mice at 12 weeks of age (*n* = 4–5). **c** Detection of miR-375 and 28S rRNA in pancreatic tissue sections from wildtype (WT), miR-375KO, and β-Rescue mice using miRNA FISH. *Green*: miR-375, *Red*: 28S rRNA, *Blue*: cell nuclei. All data shown are mean ± s.e.m. *p < 0.05, ***p < 0.005

To investigate the specific role of miR-375 in pancreatic β-cells for the metabolic impairment in the global miR-375KO mice, we crossed Tg375 mice with miR-375KO animals in order to re-constitute miR-375 expression selectively in β-cells, while leaving other islet endocrine cells and organs depleted of the miRNA (Tg375/mir-375KO mice are referred to as “β-Rescue” mice). Quantitative PCR (qPCR) indicated that expression of miR-375 was recovered to ≈85 % of wildtype (WT) in β-Rescue islets (Fig. 2a). To further validate the selective reconstitution of miR-375 levels in β-Rescue mice, we measured mRNA levels of HuD, encoding an RNA-binding protein that regulates translation of the insulin2 mRNA [21] and an evolutionarily conserved and experimentally validated miR-375 target in β-cells [10]. This analysis revealed that HuD is upregulated ≈3-fold in miR-375KO islets but downregulated in β-Rescue mice to similar levels than WT animals (Fig. 2b). Finally, in situ hybridization with miR-375 probes confirmed restoration of miR-375 expression in the core of pancreatic islets of β-Rescue mice (Fig. 2c). Together, these results show that the Tg375 transgene could selectively and functionally restore endogenous miR-375 expression in β-cells of miR-375KO mice.

**Fig. 3:**
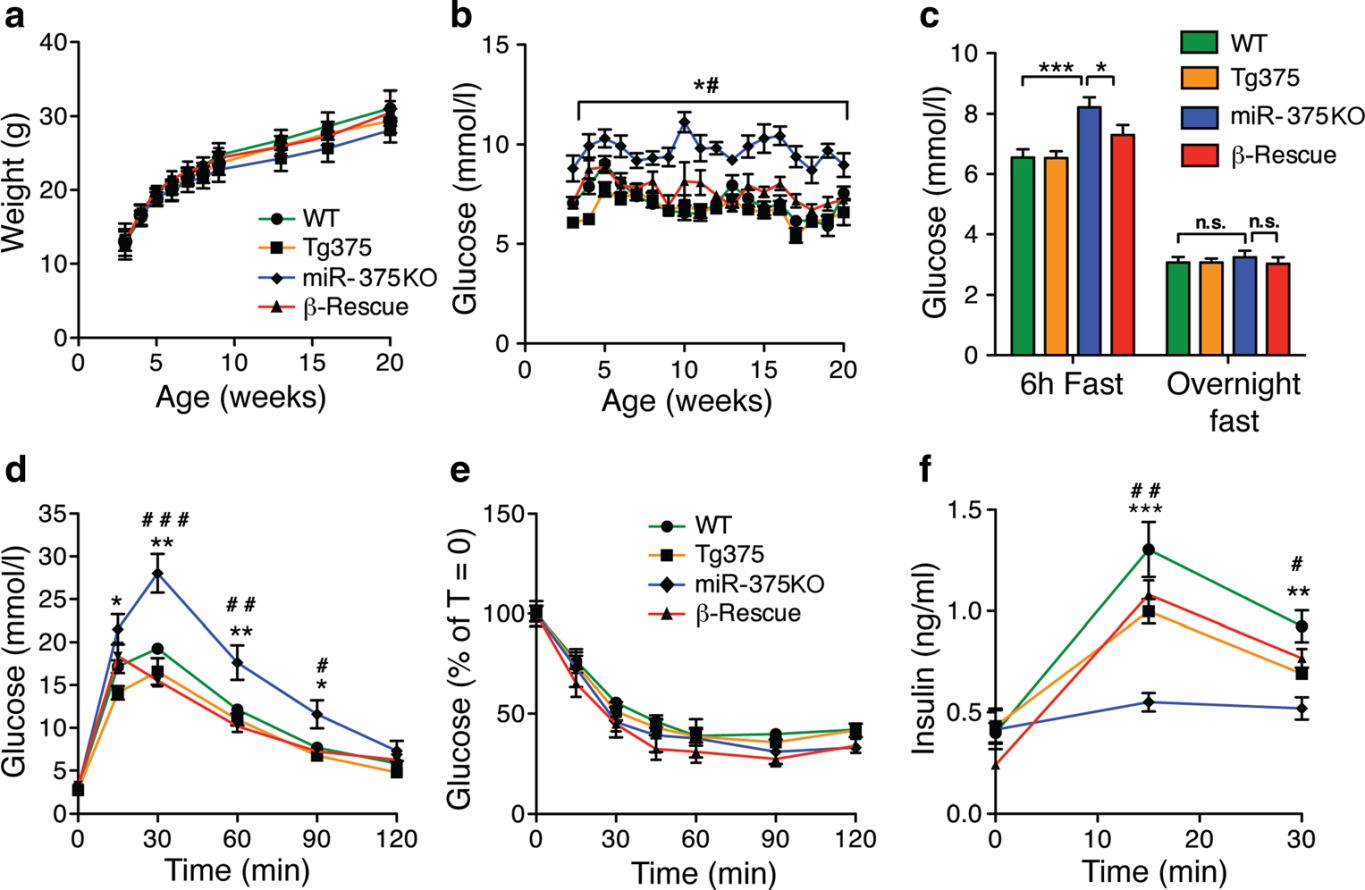
Metabolic characterization of miR-375 β-Rescue mice. WT, Tg375, miR-375KO, and β-Rescue mice were subjected to the following measurements: **a** body weight, **b** ad libitum-fed blood glucose levels, **c** fasting glucose (6 h and overnight) levels at 12 weeks of age, **d** IPGTT (2 g/kg) after an overnight fast at 13 weeks of age, **e** ITT (0.75U/kg) after an overnight fast at 11 weeks of age, **f** insulin excursion analysis after an overnight fast at 16 weeks of age (3 g/kg). All data shown are mean ± s.e.m; *n* = 6–9; **p < 0.01, ***p < 0.005 WT vs. miR-375KO, ^#^ *p* < 0.05, ^##^ *p* < 0.01, β-Rescue vs. miR-375KO

Metabolic characterization of miR-375KO and β-Rescue mice revealed no difference in body weight (Fig. 3a) or food and water intake (data not shown). As expected, miR-375KO animals developed random fed (Fig. 3b) and fasting hyperglycemia (Fig. 3c) from weaning (3 weeks of age) throughout adult life [10]. Interestingly, β-Rescue mice displayed normal blood glucose levels in fed and fasting conditions and were virtually indistinguishable from control mice (Fig. 3b, c). IPGTT revealed marked glucose intolerance in miR-375KO animals, whereas β-Rescue mice showed a glucose response similar to WT littermates (Fig. 3d). Insulin tolerance tests were similar in WT, Tg375, miR-375KO, and β-Rescue mice, indicating that insulin sensitivity was unchanged (Fig. 3e). Measurements of insulin excursions in response to glucose injection showed a profound impairment in insulin secretion at 15 min after glucose injection in miR-375KO mice (0.55 ± 0.05 ng/ml in miR-375KO vs 1.30 ± 0.14 ng/ml in WT, *p* < 0.005), which could be restored to almost WT levels in β-Rescue mice (1.30 ± 0.14 ng/ml in WT versus 1.08 ± 0.07 ng/ml in β-Rescue *p* < 0.01) (Fig. 3f). Together, these findings indicate that selective re-expression of miR-375 in β-cell of miR-375KO mice is sufficient to restore normal glycemic control.

**Fig. 4:**
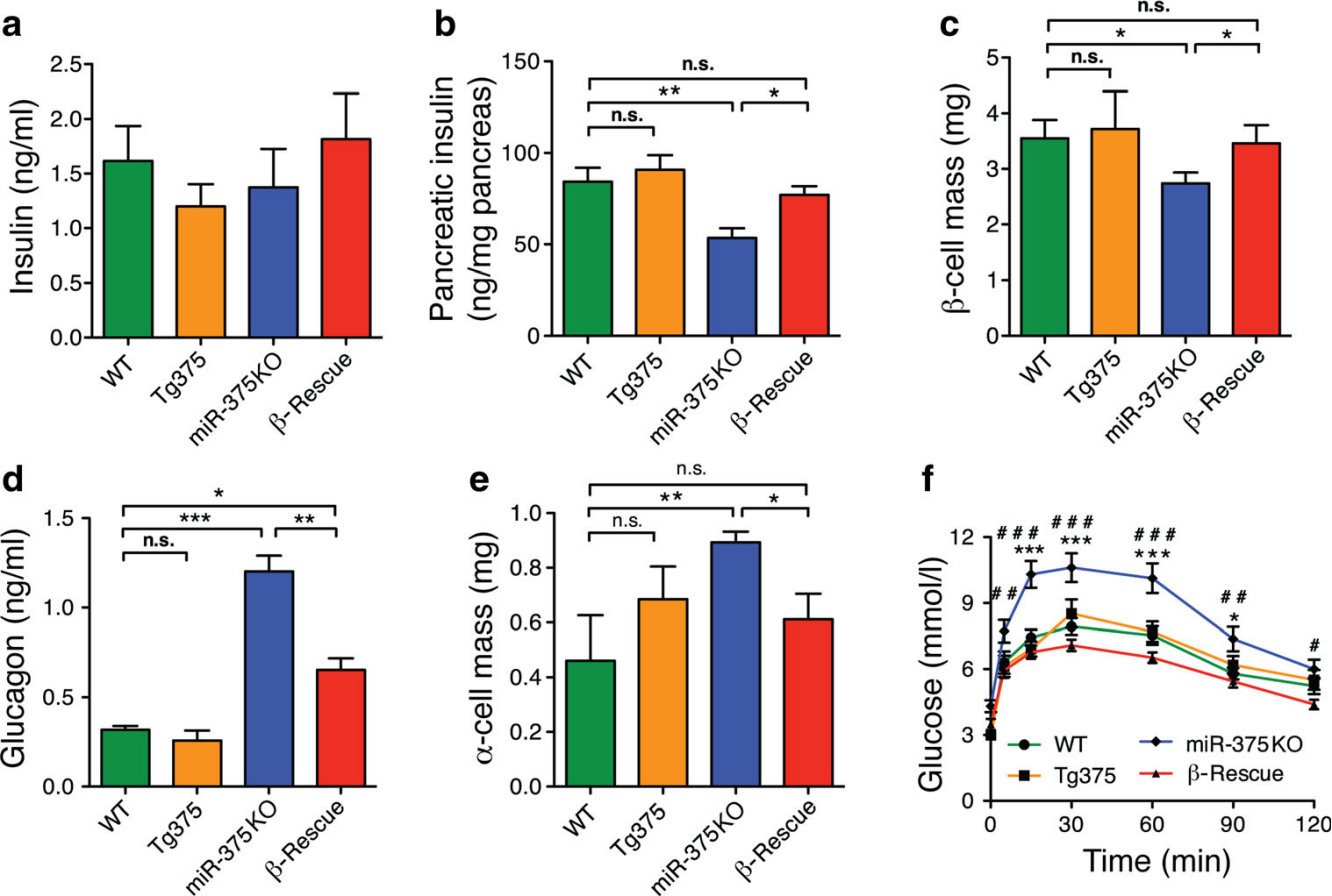
Hormone levels and islet cell mass in miR-375 β-Rescue mice. WT, Tg375, miR-375KO, and β-Rescue mice were subjected to the following measurements after a 6-h fast: **a** circulating plasma insulin levels and **b** pancreatic insulin content at 24 weeks of age (*n* = 5–6), **c** normalized pancreatic α-cell at 5 weeks of age (*n* = 5–7), **d** plasma glucagon levels in mice fasted for 3 h at 24 weeks of age (*n* = 5–6), **e** pancreatic β-cell mass at 5 weeks of age (*n* = 3–5), **f** intraperitoneal PTT in mice fasted overnight at 15 weeks of age (2 g/kg) (*n* = 6–9). All data shown are mean ± s.e.m; *p < 0.05, **p < 0.01, ***p < 0.005 WT vs. miR-375KO, ^#^ *p* < 0.05, ^##^ *p* < 0.01, ^###^ *p* < 0.005, β-Rescue versus miR-375KO

To investigate the mechanism by which the miR-375 rescue in β-cells of miR-375KO mice improved glucose tolerance, we measured pancreatic endocrine function and cell mass in these mice. Fasting insulin levels were not significantly different between miR-375KO, β-Rescue, Tg375, and WT control mice (Fig. 4a). As reported previously, we measured a decrease in pancreatic insulin content in miR-375KO mice, which was associated with reduced β-cell mass in miR-375KO mice (Fig. 4b, c) [10]. In contrast, β-Rescue mice showed partial restoration of pancreatic insulin content and β-cell mass (Fig. 4b, c). Circulating glucagon levels were increased ≈4-fold in miR-375KO mice compared to WT controls (Fig. 4d). Interestingly, reconstitution of miR-375 expression in β-cells of miR-375KO mice also decreased plasma glucagon levels compared to miR-375KO mice (Fig. 4d). Morphometric analysis revealed partial normalization of α-cell mass in β-Rescue animals (Fig. 4e). To show that selective expression of miR-375 in β-cells of miR-375KO mice is sufficient for the regulation of glucagon signaling in the liver, pyruvate tolerance was examined in mutant mice. As depicted in Fig. 4f, miR-375KO mice displayed augmented gluconeogenesis, as shown by higher blood glucose levels in response to a tolerance test (PTT). In contrast, β-Rescue mice displayed hepatic pyruvate conversion kinetics similar to those of WT controls. These results indicate that β-cell miR-375 deficiency is the primary cause underlying the phenotype of global miR-375KO mice and that the increase in α-cell mass and glucagon secretion arises secondarily to the β-cell defect.

### Release of miR-375 from pancreatic β-cells into circulation

**Fig. 5:**
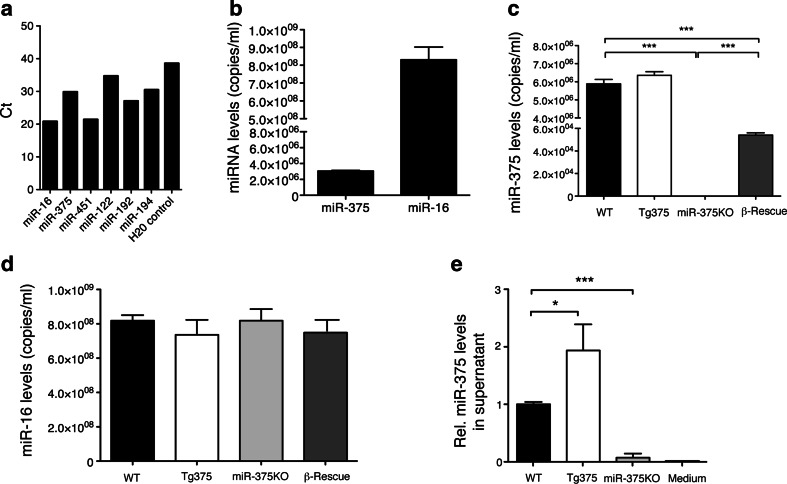
Pancreatic islets secrete miR-375 in the circulation. **a** Detection of miRNAs in C57BL/6 mouse plasma. Shown is the mean Ct value of indicated miRNAs evaluated by qPCR after 45 cycles (*n* = 3). **b** Copy number of circulating miR-375 and miR-16 in C57BL/6 mice at 7 weeks. **c**, **d** miR-375 copy number per ml of plasma of wildtype (WT), Tg375, miR-375KO, and β-Rescue mice at 20 weeks of age. **e** Relative miR-375 levels in supernatant of pancreatic islets isolated from WT, miR-375KO, and Tg375 mice cultured in serum-free media for 16 h at 37 °C. Media, serves as a negative control (*n* = 3). Data expressed as fold-change over WT controls. All data shown are mean ± s.e.m; *p < 0.05, ***p < 0.005

Several studies have suggested that miRNA expressed in β-cells are also found in the circulation [17]. However, whether these circulating miRNA indeed originate from β-cells and not from other organs still remains to be determined. To address this, we took advantage of our β-cell-specific miR-375 expressing mouse model to measure the contribution of β-cells to circulating miR-375 levels in vivo. We first measured levels of metabolically relevant miRNAs in mouse plasma. Quantitative PCR analyses revealed that miR-375 and the broadly expressed miR-16 are both readily detected in the circulation of C57BL/6 mice (20.8 ± 0.3 Ct, *n* = 3, Fig. 5a). In addition, we found that liver-enriched miR-122 and miR-192/194 as well as erythrocyte-enriched miR-451 levels were also detected in the plasma of these mice (miR-122, 34.69 ± 0.07 Ct; miR-192, 27.06 ± 0.17 Ct; miR-194, 30.48 ± 0.10 Ct; miR-451, 21.42 ± −0.16 Ct). Using an absolute quantification, we found that C56BL/6 mice presented 2 × 10^6^ miR-375 copies/ml of plasma at 7 weeks of age, while that of miR-16 was ≈400-fold higher compared to miR-375 (Fig. 5b). To determine if plasma miR-375 originates from pancreatic β-cells, we took advantage of our β-Rescue mouse model displaying selective expression of endogenous miR-375 levels in pancreatic β-cells and performed quantitative measurements in mouse plasma. These studies revealed that plasma miR-375 levels in β-Rescue mice were ≈1 % compared to WT littermates. Serum miR-375 levels in miR-375KO mice were undetectable, thus demonstrating the specificity of the qPCR assay (Fig. 5c). In contrast, plasma miR-16 levels were not different between WT, miR-375KO, and β-Rescue animals (Fig. 5d). These results reveal that a small but significant proportion of circulating miRNAs indeed is derived from pancreatic β-cells. To substantiate these findings, we performed in vitro experiments on pancreatic islets from miR-375KO, WT and Tg375 mice displaying increasing miR-375 gene dosage (Fig. 5e). After culture of pancreatic islets for 16 h in serum-free media, islet supernatants were recovered, centrifuged, and miR-375 levels quantified by qPCR. The data in Fig. 5e show that supernatant miR-375 levels correlate with islet miR-375 gene dosage. Tg375 islets secreted 1.9 ± 0.45-fold more miR-375 than WT islets, whereas miR-375 levels were virtually absent in the supernatant of miR-375KO islets (Fig. 5e). Together, these results indicate that the β-cell-enriched miR-375 is secreted from pancreatic islets, but miRNA release from this organ contributes only a small fraction to the overall blood levels in mice.

### Increased plasma levels of miR-375 in response to β-cell destruction

**Fig. 6:**
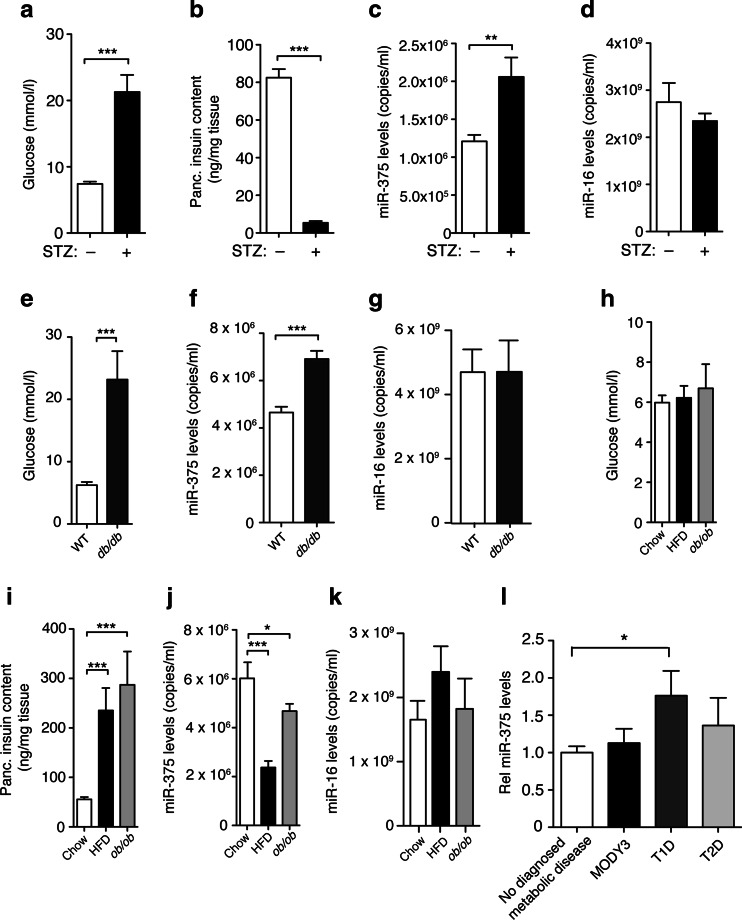
Correlation between circulating miR-375 levels and β-cell injury. **a** Blood glucose levels and **b** pancreatic insulin content in 6-h fasted C57BL/6 (10-week-old) after 72 h treatment with streptozotocin (STZ, 1 × 150 mg/kg) (*n* = 7–8). **c** Circulating miR-375 and **d** miR-16 copy number in plasma of 6-h fasted C57BL/6 WT mice (10-week-old) after being injected with STZ (+, 1 × 150 mg/kg) or PBS as control (−) for 3 days (*n* = 7–8). **e** Blood glucose and **f** circulating miR-375 and **g** miR-16 levels in WT and *db/db* (BKS-background) male mice at 8 weeks of age (*n* = 4–5). **h** Blood glucose, **i** pancreatic insulin content, and **j** circulating miR-375 and **k** miR-16 in C57BL/6 (WT) mice fed a normal or high-fat diet (HFD, for 25 weeks) and *ob/ob* (C57BL/6 background) mice (23-week-old) (*n* = 5). **l** Circulating miR-375 levels in healthy or no diagnosed metabolic disease patients (*n* = 51), HNF1α/MODY3 mutation carriers (*n* = 47), T1D (*n* = 38) and T2D (*n* = 58). All data shown are mean ± s.e.m; *p < 0.05, **p < 0.01, ***p < 0.005

Since our findings indicate that circulating miRNAs are released from β-cells, we hypothesized that their levels may correlate with β-cell mass and may serve as a biomarker of β-cell viability in diabetes. To investigate this, we employed different models of β-cell stress: an acute β-cell toxicity model induced by treating mice with STZ (150 mg/kg), obesity mouse models displaying normoglycemia due to increased β-cell proliferation, function and pancreatic insulin content (i.e., dietary (HFD), and genetic (*ob/ob* mice on C57BL/6N background) [3, 22, 23], as well as diabetic models (*db/db* mice on BLKS background) exhibiting profound hyperglycemia, reduced plasma insulin levels due to β-cell dysfunction [24, 25] and apoptosis [26, 27]. For the acute toxicity model, blood was analyzed for glucose and serum miRNAs levels 3 days after STZ injection. STZ treatment resulted in elevation of blood glucose levels, which correlated with depletion of β-cells as revealed by pancreatic insulin content measurements (Fig. 6a, b). We observed that circulating miR-375 levels were increased by ≈2-fold in STZ-treated diabetic mice as compared to controls (Fig. 6c), while those of miR-16, a ubiquitously expressed miRNA was unaffected by STZ treatment (Fig. 6d). Interestingly, increased plasma miR-375 levels were also measured in hyperglycemic BKS.*db/db* mice compared to control littermates, whereas miR-16 levels were similar (Fig. 6e–g). In contrast, our analysis revealed that miR-375 levels were decreased by ≈50 and 20 % in islets of normoglycemic HFD and *ob/ob* mice compared to lean littermate controls, respectively (Fig. 6h–k). Together, these results indicate that miR-375 levels in the circulation do not correlate with β-cell function or mass but may be a surrogate marker for β-cell injury and cell death.

Lastly, we extended our findings to diabetic patients and measured circulating miR-375 levels in human subjects with inactivating mutations in HNF1α (maturity-onset of the young, type 3 (MODY3)), T2D, T1D, and healthy subjects (no diagnosed metabolic disease (NDMD)). This analysis revealed that circulating miR-375 levels were unaltered in MODY3 and T2D but elevated in T1D subjects compared to healthy controls (Fig. 6l). Although not reaching statistical significance due to reduced patient number, we observed an overall trend toward higher levels in both genders (NDMD males 6.9 × 10^6^ ± 0.9 × 10^6^ copies/ml and T1D males 9.6 × 10^6^ ± 1.7 × 10^6^ copies/ml *n* = 16–23; NDMD females 6.8 × 10^6^ ± 0.9 × 10^6^ copies/ml; T1D females 12.5 × 10^6^ ± 2.7 × 10^6^, *n* = 22–28). Together, these results indicate that plasma miR-375 levels do not discriminate between different forms of T2D but may be used as an indicator of acute β-cell destruction and autoimmune diabetes.

## Discussion

Previous work from our group revealed that miR-375 is required for maintenance of α- and β-cell mass in mice [10]. Using genetic loss of function experiments, we found that genetic inactivation of miR-375 decreases β-cell mass but concomitantly increases α-cell mass. We now report the generation of a miR-375 gain-of-function mouse model with selective overexpression of miR-375 in pancreatic β-cells. We initially hypothesized that Tg375 mice would present improved metabolic functions associated with an increase in β-cell mass. Surprisingly, we did not observe any changes in islet endocrine cell mass, pancreatic β-cell function, and glycemic control in Tg375 mice. This could be explained by the high endogenous miR-375 levels in pancreatic islets of both mouse and human [7, 10, 28], where miR-375 mRNA targets regulating β-cell homeostasis are fully engaged and repressed by this miRNA. Therefore, any further increase in miR-375 levels is unlikely to influence target expression. For example, the levels of HuD mRNA are unchanged in Tg375 islets but found at higher levels in miR-375KO islets (Figs. 1 and 2). However, when islet miR-375 gene dosage is decreased in islets of Tg375 through inactivation of endogenous alleles of the miRNA (β–Rescue mice), the repressive action of miR-375 on HuD mRNA is recovered. These results support recent quantitative studies showing that miRNA activity is determined by a delicate balance between miRNA and mRNA target abundance [29]. Therefore, the protective role of miR-375 overexpression on β cell growth might become more important in models of pancreatic β-cells failure where loss of β-cell function represents the main driving force of metabolic dysfunction. Thus, therapeutic approaches aimed at increasing β-cell growth and proliferation through upregulation of miR-375 function should be considered only in a setting of reduced miR-375 levels.

To assess the relative importance of α- and β-cell mass remodeling for the overall phenotype of global miR-375KO mice, we conducted genetic rescue experiments using Tg375 mice and global miR-375KO mice (Fig. 3). Crossing of both mouse lines allowed us to generate mice expressing miR-375 exclusively in pancreatic β-cells. Interestingly, these β-Rescue mice were indistinguishable from wildtype animals in several aspects, including: (1) miR-375 levels in islet, (2) insulin and glucagon levels, (3) glucose, insulin and pyruvate tolerance, and (4) α- and β-cell mass (Figs. 3 and 4). These data indicate that the primary defect of global miR-375KO mice is caused by the loss of miR-375 in pancreatic β-cells, which results in a secondary and indirect α-cell growth and proliferative response. The low hypothalamic expression detected in Tg375 mice is unlikely to be responsible for the β-cell rescue phenotype since insulin secretion and glucose tolerance is indistinguishable from WT animals. It was previously shown that β-cell destruction by low doses of STZ leads to α-cell hyperplasia, partially phenocopying the β-cell hypoplasia and α-cell hyperplasia of miR-375KO mice [10, 30]. The genetic rescue experiment further demonstrates that the content of miR-375 in β-cells can indirectly influence the function and growth of α-cells. For instance, insulin is known to inhibit glucagon secretion, and the increased β-cell mass and function in β-Rescue mice might contribute to the reduced glucagon levels in these mice [31]. Direct genetic evidence for a role of β-cell derived insulin in the modulation of α-cell responses was provided by Kawamori et al. who demonstrated that insulin binding to its receptor on α-cells modulates glucagon secretion (but not α-cell mass), a coupling that is lost in obesity-induced insulin resistance and that contributes to the hyperglucagonemia measured in patients with T2D [32–34].

Recent reports have revealed that circulating miRNA expression is altered in pathological settings and may be used as biomarkers. Previous studies reported that miR-375 levels correlate with advanced prostate [35] and hepatocellular carcinoma [36] in humans and revealed to be among the most highly differentially regulated blood miRNAs in apoE-deficient mice [14]. These studies are difficult to reconcile considering the selective expression of miR-375 in neuroendocrine organs [6] and intestinal goblet cells [37] and may indicate that metabolic effects influence the secretion, clearance, or stability of miR-375. We now provide the first evidence for secretion of miR-375 by pancreatic β-cells in vivo in unstressed conditions (Fig. 5). Circulating miR-375 levels in mice exclusively expressing miR-375 in β-cells corresponded to approximately 1 % of WT mice. This indicates that although β-cells contribute to the circulating levels of miR-375, most of the miRNA originates from other organs, most likely neuroendocrine cells from lung, gastrointestinal tract, thyroid, and adrenals. We found increased circulating miR-375 levels in response to acute pancreatic β-cell injury induced by STZ, indicating that acute β-cell death can result in increased circulating miR-375 levels. These data are in accordance with Erener et al. reporting increased miR-375 levels in STZ-treated and NOD mice and islets [18]. In agreement with this study, we also found increased circulating miR-375 levels in a mouse model of BKS-*db/db* mice that display β-cell apoptosis and profound hyperglycemia. In contrast, plasma miR-375 levels were reduced in two normoglycemic models of obesity (HFD and *ob/ob* on C57BL/6 background) with increased pancreatic β-cell function, proliferation and absence of apoptosis. This reduction in plasma miR-375 levels may be due to increased renal clearance, since miRNAs are secreted in the urine, and body weight is positively correlated with glomerular filtration rate [38, 39], and an inverse correlation exists between miRNAs abundance and kidney function [40]. This may also explain why miR-375 levels were not significantly increased in our T2D cohort in contrast to what was recently reported by Higuchi et al. where patients exhibits a much higher BMI (31.6) than the cohort we analyzed (BMI = 25.9) [41]. It will therefore be interesting to measure miR-375 urinary excretion in obese and non-obese subjects with normal and impaired glucose tolerance. Plasma miR-375 therefore is unlikely to serve as a marker of altered β-cell function, a notion also supported by the similar plasma miR-375 levels in subjects with MODY3 that is known to exhibit β-cell dysfunction. Importantly, our quantitative finding that release of miR-375 from living β-cells in vivo only contributes a very small fraction to the total plasma levels offers a rational explanation why changes in β-cell function and mass are insufficient to translate into measurable alterations in miR-375 plasma levels.

While diagnostic approaches for T1D have been developed, there is a need to identify novel predictive molecules to identify and monitor disease progression with greater accuracy. To our surprise, our data indicate that circulating miR-375 levels are increased in human T1D. Considering the small contribution of β-cells to miR-375 levels in the blood, we believe that the most likely explanation for this observation is that hyperglycemia per se elicits increased miR-375 secretion from tissues other than pancreatic β-cells. Alternatively, increased miR-375 levels in the plasma of T1D may result in reduced renal clearance, a notion that is supported by the correlation of urinary miR-21 levels with the rate of kidney function decline and risk of progression to dialysis-dependent kidney failure [40, 42]. Lastly, it is possible that continuous autoimmune destruction and regeneration of β-cells in T1D may contribute to increased plasma miR-375 levels. This is further supported by plasma miRNA profiling in children with newly diagnosed T1D that revealed elevated levels of twelve highly expressed miRNAs [43]. Increased α-cell mass observed in human T1D patients could also contribute to the elevated miR-375 levels in the circulation in the absence of β-cells [44–46].

## Electronic supplementary material

ESM 1 (PDF 121 kb)

## References

[1] Pirot P, Cardozo AK, Eizirik DL (2008). Mediators and mechanisms of pancreatic beta-cell death in type 1 diabetes. Arq Bras Endocrinol Metabol.

[2] Kahn SE, Hull RL, Utzschneider KM (2006). Mechanisms linking obesity to insulin resistance and type 2 diabetes. Nature.

[3] Prentki M, Nolan CJ (2006). Islet beta cell failure in type 2 diabetes. J Clin Invest.

[4] Garber AJ (2011). Incretin effects on β-cell function, replication, and mass: the human perspective. Diabetes Care.

[5] Ebert MS, Sharp PA (2012). Roles for microRNAs in conferring robustness to biological processes. Cell.

[6] Landgraf P, Rusu M, Sheridan R, Sewer A, Iovino N, Aravin A, Pfeffer S, Rice A, Kamphorst AO, Landthaler M (2007). A mammalian microRNA expression atlas based on small RNA library sequencing. Cell.

[7] Poy MN, Eliasson L, Krutzfeldt J, Kuwajima S, Ma X, Macdonald PE, Pfeffer S, Tuschl T, Rajewsky N, Rorsman P (2004). A pancreatic islet-specific microRNA regulates insulin secretion. Nature.

[8] Avnit-Sagi T, Vana T, Walker MD (2012). Transcriptional mechanisms controlling miR-375 gene expression in the pancreas. Exp Diabetes Res.

[9] Avnit-Sagi T, Kantorovich L, Kredo-Russo S, Hornstein E, Walker MD (2009). The promoter of the pri-miR-375 gene directs expression selectively to the endocrine pancreas. PLoS ONE.

[10] Poy MN, Hausser J, Trajkovski M, Braun M, Collins S, Rorsman P, Zavolan M, Stoffel M (2009). miR-375 maintains normal pancreatic alpha- and beta-cell mass. Proc Natl Acad Sci U S A.

[11] Cortez MA, Calin GA (2009). MicroRNA identification in plasma and serum: a new tool to diagnose and monitor diseases. Expert Opin Biol Ther.

[12] Zubakov D, Boersma AW, Choi Y, van Kuijk PF, Wiemer EA, Kayser M (2010). MicroRNA markers for forensic body fluid identification obtained from microarray screening and quantitative RT-PCR confirmation. Int J Legal Med.

[13] Valadi H, Ekstrom K, Bossios A, Sjostrand M, Lee JJ, Lotvall JO (2007). Exosome-mediated transfer of mRNAs and microRNAs is a novel mechanism of genetic exchange between cells. Nat Cell Biol.

[14] Vickers KC, Palmisano BT, Shoucri BM, Shamburek RD, Remaley AT (2011). MicroRNAs are transported in plasma and delivered to recipient cells by high-density lipoproteins. Nat Cell Biol.

[15] Arroyo JD, Chevillet JR, Kroh EM, Ruf IK, Pritchard CC, Gibson DF, Mitchell PS, Bennett CF, Pogosova-Agadjanyan EL, Stirewalt DL (2011). Argonaute2 complexes carry a population of circulating microRNAs independent of vesicles in human plasma. Proc Natl Acad Sci.

[16] Li L, Zhu D, Huang L, Zhang J, Bian Z, Chen X, Liu Y, Zhang CY, Zen K (2012). Argonaute 2 complexes selectively protect the circulating microRNAs in cell-secreted microvesicles. PLoS ONE.

[17] Guay C, Regazzi R (2013). Circulating microRNAs as novel biomarkers for diabetes mellitus. Nat Rev Endocrinol.

[18] Erener S, Mojibian M, Fox JK, Denroche HC, Kieffer TJ (2013). Circulating miR-375 as a biomarker of β-cell death and diabetes in mice. Endocrinology.

[19] Renwick N, Cekan P, Masry PA, McGeary SE, Miller JB, Hafner M, Li Z, Mihailovic A, Morozov P, Brown M (2013). Multicolor microRNA FISH effectively differentiates tumor types. J Clin Invest.

[20] Kroh EM, Parkin RK, Mitchell PS, Tewari M (2010). Analysis of circulating microRNA biomarkers in plasma and serum using quantitative reverse transcription-PCR (qRT-PCR). Methods.

[21] Lee EK, Kim W, Tominaga K, Martindale JL, Yang X, Subaran SS, Carlson OD, Mercken EM, Kulkarni RN, Akamatsu W (2012). RNA-binding protein HuD controls insulin translation. Mol Cell.

[22] Bleisch VR, Mayer J, Dickie MM (1952). Familial diabetes mellitus in mice, associated with insulin resistance, obesity, and hyperplasia of the islands of langerhans. Am J Pathol.

[23] Hull RL, Kodama K, Utzschneider KM, Carr DB, Prigeon RL, Kahn SE (2005). Dietary-fat-induced obesity in mice results in beta cell hyperplasia but not increased insulin release: evidence for specificity of impaired beta cell adaptation. Diabetologia.

[24] Latreille M, Hausser J, Stutzer I, Zhang Q, Hastoy B, Gargani S, Kerr-Conte J, Pattou F, Zavolan M, Esguerra JL (2014). MicroRNA-7a regulates pancreatic beta cell function. J Clin Invest.

[25] Do OH, Low JT, Gaisano HY, Thorn P (2014). The secretory deficit in islets from db/db mice is mainly due to a loss of responding beta cells. Diabetologia.

[26] Puff R, Dames P, Weise M, Goke B, Seissler J, Parhofer KG, Lechner A (2011). Reduced proliferation and a high apoptotic frequency of pancreatic beta cells contribute to genetically-determined diabetes susceptibility of db/db BKS mice. Horm Metab Res.

[27] Dalboge LS, Almholt DL, Neerup TS, Vassiliadis E, Vrang N, Pedersen L, Fosgerau K, Jelsing J (2013). Characterisation of age-dependent beta cell dynamics in the male db/db mice. PLoS ONE.

[28] Pullen TJ, da Silva XG, Kelsey G, Rutter GA (2011). miR-29a and miR-29b contribute to pancreatic beta-cell-specific silencing of monocarboxylate transporter 1 (Mct1). Mol Cell Biol.

[29] Mullokandov G, Baccarini A, Ruzo A, Jayaprakash AD, Tung N, Israelow B, Evans MJ, Sachidanandam R, Brown BD (2012). High-throughput assessment of microRNA activity and function using microRNA sensor and decoy libraries. Nat Methods.

[30] Li Z, Karlsson FA, Sandler S (2000). Islet loss and alpha cell expansion in type 1 diabetes induced by multiple low-dose streptozotocin administration in mice. J Endocrinol.

[31] Hamaguchi K, Utsunomiya N, Takaki R, Yoshimatsu H, Sakata T (2003). Cellular interaction between mouse pancreatic α-cell and β-cell lines: possible contact-dependent inhibition of insulin secretion. Exp Biol Med.

[32] Asplin CM, Paquette TL, Palmer JP (1981). In vivo inhibition of glucagon secretion by paracrine beta cell activity in man. J Clin Invest.

[33] Maruyama H, Hisatomi A, Orci L, Grodsky GM, Unger RH (1984). Insulin within islets is a physiologic glucagon release inhibitor. J Clin Invest.

[34] Kawamori D, Kurpad AJ, Hu J, Liew CW, Shih JL, Ford EL, Herrera PL, Polonsky KS, McGuinness OP, Kulkarni RN (2009). Insulin signaling in alpha cells modulates glucagon secretion in vivo. Cell Metab.

[35] Brase JC, Johannes M, Schlomm T, Falth M, Haese A, Steuber T, Beissbarth T, Kuner R, Sultmann H (2011). Circulating miRNAs are correlated with tumor progression in prostate cancer. Int J Cancer.

[36] Li LM, Hu ZB, Zhou ZX, Chen X, Liu FY, Zhang JF, Shen HB, Zhang CY, Zen K (2010). Serum microRNA profiles serve as novel biomarkers for HBV infection and diagnosis of HBV-positive hepatocarcinoma. Cancer Res.

[37] Biton M, Levin A, Slyper M, Alkalay I, Horwitz E, Mor H, Kredo-Russo S, Avnit-Sagi T, Cojocaru G, Zreik F (2011). Epithelial microRNAs regulate gut mucosal immunity via epithelium-T cell crosstalk. Nat Immunol.

[38] Bosma RJ, van der Heide JJ, Oosterop EJ, de Jong PE, Navis G (2004). Body mass index is associated with altered renal hemodynamics in non-obese healthy subjects. Kidney Int.

[39] Chagnac A, Weinstein T, Herman M, Hirsh J, Gafter U, Ori Y (2003). The effects of weight loss on renal function in patients with severe obesity. J Am Soc Nephrol.

[40] Neal CS, Michael MZ, Pimlott LK, TYong TY, Li JYZ, Gleadle JM (2011). Circulating microRNA expression is reduced in chronic kidney disease. Nephrol Dial Transplant.

[41] Higuchi C, Nakatsuka A, Eguchi J, Teshigawara S, Kanzaki M, Katayama A, Yamaguchi S, Takahashi N, Murakami K, Daisuke Ogawa D (2015). Identification of circulating miR-101, miR-375 and miR-802 as biomarkers for type 2 diabetes. Metabolism.

[42] Szeto CC, Ching-Ha KB, Ka-Bik L, Mac-Moune LF, Cheung-Lung CP, Gang W, Kai-Ming C, Kam-Tao LP (2012). Micro-RNA expression in the urinary sediment of patients with chronic kidney diseases. Dis Markers.

[43] Nielsen LB, Wang C, Sorensen K, Bang-Berthelsen CH, Hansen L, Andersen ML, Hougaard P, Juul A, Zhang CY, Pociot F (2012). Circulating levels of microRNA from children with newly diagnosed type 1 diabetes and healthy controls: evidence that miR-25 associates to residual beta-cell function and glycaemic control during disease progression. Exp Diabetes Res.

[44] Gepts W, De Mey J (1978). Islet cell survival determined by morphology. An immunocytochemical study of the islets of Langerhans in juvenile diabetes mellitus. Diabetes.

[45] Gepts W, Lecompte PM (1981). The pancreatic islets in diabetes. Am J Med.

[46] Somoza N, Vargas F, Roura-Mir C, Vives-Pi M, Fernandez-Figueras MT, Ariza A, Gomis R, Bragado R, Marti M, Jaraquemada D (1994). Pancreas in recent onset insulin-dependent diabetes mellitus. Changes in HLA, adhesion molecules and autoantigens, restricted T cell receptor V beta usage, and cytokine profile. J Immunol.

