# Supplementary material for: miR-375 gene dosage in pancreatic β-cells: implications for regulation of β-cell mass and biomarker development
Source: J Mol Med (Berl). 2015 May 28;93(10):1159–69. doi: 10.1007/s00109-015-1296-9 (PMC4589563; doi:10.1007/s00109-015-1296-9)
Supplement: Supplementary file 1 — (PDF 121 kb) [file 109_2015_1296_MOESM1_ESM.pdf]

Electronic supplementary material (ESM)

J Mol Med 2015

miR-375 gene dosage in pancreatic β-cells: Implications for regulation of β-cell mass and biomarker development

Mathieu Latreille[1,2], Karolin Herrmanns[1] , Neil Renwick[3], Thomas Tuschl[3], Maciej T. Malecki[4,5], Mark I. McCarthy[6,7,8], Katharine R. Owen[8], Thomas Rülicke[9] and Markus Stoffel[1,10]

[1] Institute of Molecular Health Sciences, ETH Zurich, Otto-Stern Weg. 7, 8093 Zurich, Switzerland
[2] Current address: MRC Clinical Sciences Centre, Imperial College London, Hammersmith Hospital Campus Du Cane Road, London W12 0NN
[3] Howard Hughes Medical Institute, Laboratory of RNA Molecular Biology, The Rockefeller University, 1230 York Avenue, Box 186, New York, NY 10065, USA
[4] Department of Metabolic Diseases, Jagiellonian University Medical College, Krakow, Poland
[5] University Hospital, Krakow, Poland
[6] Oxford Centre for Diabetes, Endocrinology and Metabolism, University of Oxford, Churchill Hospital, Old Road, Headington, Oxford, OX3 7LJ, UK
[7] Wellcome Trust Centre for Human Genetics, University of Oxford, Roosevelt Drive, Oxford OX3 7BN, UK
[8] Oxford NIHR Biomedical Research Centre, Churchill Hospital, Old Road, Headington, Oxford, OX3 7LJ, UK
[9] Institute of Laboratory Animal Science, University of Veterinary Medicine. Vienna, Austria
[10] Faculty of Medicine, University of Zurich, Zurich, Switzerland

Correspondence:
Markus Stoffel, Swiss Federal Institute of Technology (ETH Zurich), Institute of Molecular Health Sciences, Otto-Stern-Weg 7, HPL H36, 8093 Zurich, Switzerland, Tel: +41 44 633 4560, Fax: +41 44 633 1362, e-mail: stoffel@biol.ethz.ch

Supplementary Table 1:
Clinical and biochemical characteristics of subject

NDMD: no diagnosed metabolic disease (controls). Data shown are mean ± s.d. vs NDMD. *: *p*<0.05, **: *p*<0.01, ***: *p*<0.005; n.d.: not determined.

**Patient (Male/Female)**	**Age (years)**	**BMI (kg/m^2)**	**Fasting Glucose (mmol/l)**	**Hb1Ac (mmol/mol)**	**Insulin ([illegible]**
NDMD (26/25)	40.8± 5.7	24.7±2.8	5.3±0.5	n.d.	7.7±6.9
HNF1 [illegible]	36.6±15.1	23.2±4.1*	7.2±2.8 ***	6.8±1.5	7.9±6.2
T1D (16/22)	43.6±8.8	25.7±5.6	11.7±8.5 **	n.d.	n.d.
T2D (38/20)	54.2±11.2 ***	31.6±6.5***	8.3±3.2***	8.1±1.6	24.6±23.0***
